# The Immunomodulation Role of Vaginal Microenvironment On Human Papillomavirus Infection

**DOI:** 10.31661/gmj.v12i.2991

**Published:** 2023-05-20

**Authors:** Lingyan Sun, Li Li, Wenxin Xu, Cen Ma

**Affiliations:** ^1^ Department of Obstetrics and Gynecology Laboratory, The First Affiliated Hospital of Soochow University, Suzhou, Jiangsu 215000, China.

**Keywords:** Human Papillomavirus, Vaginal Microenvironment, Lactobacillus acidophilus, Cervical Cancer, Cervical Intraepithelial Neoplasia

## Abstract

Background: Evidence suggests the role of the vaginal microbiome and microenvironment in the immunity state. The human papillomavirus (HPV) infection is widely dependent on the healthy vaginal microenvironment. Hence, this study aimed to investigate the role of the vaginal microenvironment in the rate of high-risk HPV (hr-HPV) infection. Materials and Methods: This cross-sectional study was performed on 512 women with hr-HPV positive (n=212) or negative (n=300) infection. The vaginal samples of women were examined regarding yeas and Gardnerella vaginalis infection. Also, Lactobacillus acidophilus, pH, and enzyme activity (such as catalase, proline aminopeptidase, and leucocyte esterase) were compared between the two groups. Also, the histopathological study was performed on the vaginal samples.Results: The higher rate of yeast and G. vaginalis infections as well as decreased L. acidophilus, were significantly observed in women with hr-HPV positive infection (P0.001). Also, histopathological findings indicated that cervical intraepithelial neoplasia grade I-III and cervical cancer lesions were markedly higher in hr-HPV positive group compared with control women.Conclusion: The hr-HPV infection was markedly correlated to vaginal microenvironments, and it could a risk factor for the elevation of the rate of high-grade cervical lesions.

## Introduction

In the vagina of healthy women, coexist of multiple microorganisms provides the natural micro-ecological environment [1]. Vaginal dysbiosis is a common condition that disrupts the immune balance, leading to a breakdown in the protective layer of cells and enhanced sexually transmitted infections [2]. Indeed, vaginal dysbiosis is an imbalance in the types and amounts of bacteria in the vagina, which can lead to various diseases [2]. In a normal microenvironment vagina, Lactobacillus acidophilus is the main microorganism that balances the vaginal environment [3]. When the vagina is affected by exo- and/or endogenous factors, the imbalance of vaginal microecology occurs [3]. The common vaginal infectious diseases in women include bacterial vaginosis (BV), vulvovaginal candidiasis, Trichomonas vaginitis, and aerobic vaginitis (AV) [3][4]. The human papillomavirus (HPV) is a small spherical DNA virus with no envelope, which can lead to abnormal proliferation of squamous epithelium of cervical mucosa and then develop into cervical lesions and even cervical cancer (CC) [4]. The HPV infection of most susceptible people is transient and can be eliminated by itself, but when the vaginal microecology is impaired, the high-risk HPV (hr-HPV) persistent infection may occur [5]. HPV infection can cause local immune changes in the cervix and vaginal mucosa, change the composition of vaginal flora, and lead to oxidative stress reaction, which impairs the antioxidant enzyme system [6][7][8]. Chao et al. showed that some vaginitis diseases play an important role in the gradual transformation of normal cervical tissues into cancer [9]. Hence, in this study, we aimed to investigate the role of the microenvironment of the vagina on hr-HPV infection and its association with cervical lesions.

## Materials and Methods

1. Participants and Study Design

This cross-sectional study was performed on 512 women who visited at the outpatient department of Obstetrics and Gynecology of the affiliated hospital of Soochow University, Suzhou, China, during 2019-2022. All the women were evaluated regarding HPV status and divided into hr-HPV-positive group and controls (hr-HPV-negative). HPV infection diagnosis was performed based on standard methods. Briefly, the exfoliated cells were centrifuged at room temperature at 12000 R /minute for 1 minute. HPV-DNA was extracted with DNA Extraction Kit (Wuhan Boruida Biotechnology, China). HPV genotypes were detected by polymerase chain reaction based on hybridization microarray. This genotyping test can qualitatively detect 18 high-risk types (such as HPV16, 18, 31, 33, 35, 39, 45, 51, 52, 53, 56, 58, and 59) and three low-risk types (such as HPV6, 11, and 81). In this study, HPV-DNA positivity was defined as the diagnostic standard of HPV infection positivity, and HPV16, 18, 31, 33, 35, 39, 45, 51, 52, 53, 56, 58, and 59 were considered as 13 high-risk types.

2. Sample Size Calculation

Regarding Lv et al. [10], vaginal infections were observed in 0.36 % of controls, and with α=0.05 and power of 0.8, the sample size was calculated as 187 for each group.

3. Inclusion and Exclusion Criteria

All the women aged over 18 years were included in this study. Also, women with pregnancy, history of genitalia dysplasia and/or malignancy, human immunodeficiency virus (HIV) infection, immunosuppressive diseases, history of chemotherapy and/or radiotherapy, multi-partner, and history of previous HPV infection were excluded from the study.

4. Data Collection

4.1. Vaginal Sampling

To assess the vaginal microenvironment, secretions on the lateral wall of the vagina were collected with three sterile cotton swabs by trained midwives based on a similar protocol [11].

4.2. Assessment AV

One swab was used by the chemical reaction method for AV. The AV was considered positive in the case of ‎Donders diagnostic scale score ≥3 points.

4.3. Determine pH of Vaginal Secretion

The pH value of vaginal ‎secretions of the two groups was examined using a joint inspection ‎analyzer (Anhui Anchang Biotechnology, China) with the vaginitis five-link Kit (Guangzhou Ruihui Biotechnology, China), and the results showed that blue means ‎pH≥4.8, and yellow and green means pH<4.8.

4.4. Normal Flora

One swab was placed ‎into a test tube containing a small amount of 0.9% sodium chloride for ‎routine microscopic examination, including leukocytes, epithelial cells, T. vaginalis, Candida, L. acidophilus, miscellaneous bacteria, and clue cells. L. acidophilus was considered absent if its counts were ≤30/ in each high power field (HPF), and counts of >30/HPF were considered normal. Observation of candida spores and hyphae under the ‎microscope was considered positive; microscopic evaluation also detected T. vaginalis. ‎

4.5. Measurement Proline Aminopeptidase, Catalase, and Leucocyte Esterase Activity

To evaluation of the proline aminopeptidase activity, a microplate was used, and GBC fast garnet salt (Sigma, Germany) was added after 4 hours of incubation at 35°C. After an additional 5 minutes of incubation with the dye, a pink-red color indicated a positive test, while a yellowish-amber color indicated a negative test [12].

Based on the manufacturer’s instructions, Chemstrip Leucocyte esterase was used (Boeringer Mannheim Diagnostics, China). A dipstick test with a trace color was considered positive [13]. Also, catalase enzyme activity was evaluated based on changes in measured absorbance at 240 nm of wavelength.

4.6. Identification of BV

Certain criteria, such as the presence of vaginal discharge, pH >4.5, amine production, and a decrease in Lactobacillus morphotypes, were considered to diagnose BV, along with specific bacterial morphotypes.

Also, in the case of Nugent ≥7 points, the sample was considered positive for BV. Although recovering Gardnerella vaginalis by culture was not a specific indicator of BV, it was considered as additional test [14].

4.7. Histopathological Study

Cervical tissues were collected by colposcopy biopsy or cervical circumcision for histopathological examination. Two expert pathologists who were blinded to the results of high-risk HPV-DNA testing read the films and finally reported the pathological sections according to the Bethesda System classification recommended by the International Cancer Institute in 2001 [4].

Pathological examination results were divided into normal or inflammatory, cervical intraepithelial neoplasia (CIN) I-III, and CC. Normal or inflamed cervix was defined as chronic cervicitis (NC), CIN grade I was defined as a low-grade squamous intraepithelial lesion (LSIL), and CIN grade II and III were defined as high-grade SIL (HSIL).

5. Ethical Issues

This study was approved by the First Affiliated Hospital of Soochow University (approval code: 201906001). Also, the written informed constant was obtained from all the participants.

6. Statistical Analysis 

Data were presented as number and percent and/or mean and standard deviations. The IBM SPSS Statistics for Windows, version 21 (IBM Corp., Armonk, NY, USA) using the chi-square test was applied for data analysis. Also, logistic regression analysis was used for multivariate analysis to determine the Odds ratio (OR). A P-value<0.05 was considered as statistically significant.

## Results

Regarding HPV testing, 212 women had positive results, and 300 were considered HPV-negative. The median age of participants in hr-HPV-positive and -negative groups were 38 years (ranged 20-65) and 37 years (ranged 22-65).

There were no significant differences among studied women in terms of age (P=0.056). Regarding Table-1, there was no significant difference between the two groups in terms of pH (OR= 0.84, P=0.67).

However, the presence of proline aminopeptidase (OR=4.83), catalase (OR=2.41), and leucocyte esterase (OR=2.531) were significantly associated with HPV positivity (P˂0.001, Table-1). As shown in Table-2, yeast, G. vaginalis, and L. acidophilus were significantly more observed among women in hr-HPV positive group (P<0.001).

Indeed, HPV positivity reduced L. acidophilus in 70.28% of women compared with 52.33% in controls (OR: 1.71, P=0.017, Table-2).

Multivariate logistic regression analysis showed that yeast infection, G. vaginalis infection, catalase positivity, proline aminopeptidase activity, leucocyte esterase positivity, and decreased L. acidophilus were independent risk factors for hr-HPV-positive infection (P<0.05).

**Table:** Table1. The Baseline Characteristics of Studied Women

Parameters		hr-HPV	OR (95% CI)	P-value**
	Positive (n=212)	Negative (n=300)		
pH, n(%)				
‎≧4.6‎	14 (6.6)	17 (5.6)	0.84 (0.39 to 1.8)	0.673
<4.6	198 (93.4)	283 (94.4)
P-value*	0.774		
Proline aminopeptidase, n(%)				
Absent	175 (82.5)	283 (94.4)	4.83 (2.17 to 11.02)	˂0.001
Present	37 (17.5)	17 (5.6)
P-value*	0.01		
Leucocyte esterase, n(%)				
Absent	52 (24.5)	93 (31)	2.531 (1.28 to 3.65)	<0.001
Present	160 (75.5)	207 (69)
P-value*	0.323			
Catalase, n(%)				
Absent	21 (28.3)	154 (51.4)	‎2.41 (1.26 to 4.62)‎	‎0.008‎
Present	152 (71.7)	146 (48.6)
P-value*	<0.001		

^*^ Between two groups based on the Chi-square test ^**^ Logistic regression **HPV:** Human papillomavirus;**CI:**Confidence interval

**Table:** Table2. Vaginal Microbial Features of Women

Parameters		hr-HPV	OR (95% CI)	P-value**
	Positive (n=212)	Negative (n=300)		
L. acidophilus, n(%)				
Normal	63 (29.7)	143 (47.6)	1.71 (1.15 to 2.53)	0.017
Decreased	149 (70.3)	157 (52.3)
P-value*	0.009		
Yeasts, n(%)				
Absent	100 (47.1)	227 (75.6)	‎2.7 (‎1.04 to 7.03)‎	0.041
Present	112 (52.9)	73 (24.4)
P-value*	<0.001		
G. vaginalis, n(%)				
Absent	112 (52.9)	237 (79)	‎3.35 ‎(‎1.44 to 7.8)	0.005
Present	100 (47.1)	63 (21)
P-value*	<0.001		

^*^ Between two groups based on the Chi-square ^**^ Logistic regression **HPV:** Human papillomavirus;**OR:** Odds ratio;**CI:**Confidence interval

**Table:** Table3. Histopathological Findings of Studied Women

Parameters		hr-HPV	OR (95% CI)	P-value*	P-value**
	Positive (n=212)	Negative (n=300)			
NC	80 (37.74)	272 (90.6)	0.35 (0.23 to 0.53)	0.002	<0.001
LSIL	32 (15.09)	12 (4)	0.23 (0.11 to 0.46)	0.008	<0.001
HSIL	90 (42.45)	15 (5)	0.07 (0.04 to 0.12)	<0.001	<0.001
CC	10 (4.72)	1 (0.4)	0.06 (0.009 to 0.53)	0.024	0.01

^*^ Between two groups based on the Chi-square test ^**^ Logistic regression **HPV:** Human papillomavirus;**NC:** Non-cancer;**LSIL:** Low-grade squamous intraepithelial lesion;**HSIL:** High-grade squamous intraepithelial lesion;**CC:** Cervical cancer;**OR:** Odds ratio;**CI:**Confidence interval

Histopathological Study

The histopathological findings revealed that 352 (68.7%) of all the individuals had NC ‎results; however, 11 (2.1%) women have malignant lesions (Table-3). The LSIL, HSIL, and CC were significantly observed in the women of the hr-HPV positive group (P<0.001‎, Table-3). Indeed, HPV infection leads to malignant changes in the vagina and cervix of women.

## Discussion

The results of the current study showed that HPV infection was correlated with the presence of proline aminopeptidase, catalase, and leucocyte esterase. Also, high-grade lesions were significantly observed among women with hr-HPV infection.

Our findings in line with previous studies indicated that L. acidophilus was more frequent in women with HPV-negative infection [15][16][17]. In contrast, yeast and G. vaginalis were significantly present in women with HPV-positive infection. Santella et al. [18] demonstrated that the microbiota composition in patients with HPV infection differed significantly from control women.

Wei et al. [19] found that there were microbial perturbations in the early phase of hr-HPV infection, with a decrease in L. acidophilus and an increase in bacteria related to BV, such as G. vaginalis.

Their findings are consistent with our study. Also, Wei et al. [19] suggested that the predominance of some BV-associated bacteria during hr-HPV infection may increase the risk for cervical neoplasia.

In our study, the detection rates of yeasts and G. vaginalis in the hr-HPV-positive group were also higher than those in the HPV-negative group.

In line with our study, Dareng et al. [20] evaluated the association between vaginal microbiota and persistent hr-HPV infection in vaginal microbiota of Nigerian women, which showed a high diversity vaginal microbial community with a paucity of Lactobacillus species was associated with persistent hr-HPV infection.

The normal vaginal microbiota usually maintains the acidic environment in the vagina by producing lactic acid and maintains the self-purification effect of the vagina [21][22]. In the normal vaginal environment, the quantity and function of L. acidophilus play an important role in maintaining the vaginal microenvironment [22]. When the number of L. acidophilus decreases, the normal acidic environment of the vagina is impaired, and the antimicrobial effect is reduced. Pathogenic bacteria could invade the vagina and proliferate, resulting in infection of the reproductive system and changes in the microenvironment of bacteria in the vagina [23].

Indeed, reproductive system infection could lead to abnormal levels of sialidase and other enzymes and increase the risk of HPV infection. For example, G. vaginalis infection may disrupt the protective mucosal barrier, cause micro damage and/or change of epithelial cells, and increase HPV susceptibility [23]. In our study, G. vaginalis infection was more observed among women with HPV-positive infection. Hence, it seems that one of the possible roles of the microenvironment of the vagina in the protection of HPV infection is related to the increase of G. vaginalis.

Fungal infection can cause inflammatory reactions, increase tissue permeability, produce invasive enzymes, destroy epithelial cells, and increase HPV susceptibility [5]. Hydrogen peroxide has antibacterial activity, while catalase inhibits hydrogen peroxide; consequently, the susceptibility to HPV infection dramatically increased. Our results showed that the catalase activity in the HPV-positive group was markedly higher than in the control group.

The LSIL, especially in young women, is usually due to transient HPV infection, while persistent HPV infection is associated with HSIL and CC [24][25]. Our study indicated that the rate of HPV infection in patients with high-grade cervical lesions (LSIL, HSIL, CC groups) was higher than that in the NC group.

Chan et al. [26] showed that the rate of HPV-positive infection in patients with CIN grade I was significantly lower than that in patients with CIN II- III and invasive CC. In the early stage of HPV infection, the virus is generally in an active replication period, but the immune system can recognize it. However, when the vaginal environment is abnormal, and the vaginal mucosa and cervical epithelium are damaged, it may enhance the persistent infection of HPV, reduce the clearance rate of HPV, and ultimately increase the risk of high-grade CIN [27].

Limitations

In this study, we only examined some important microbial parameters, while other microbial species as well as a larger sample size, could provide better evidence regarding the exact role of the vaginal microenvironment in the increase of HPV infection.

## Conclusion

The hr-HPV infection is closely related to the vaginal microenvironment, and G. vaginalis and yeast infections play an important role in increasing the rate of HPV infection. Also, HPV infection could lead to high-grade cervical lesions and elevate the rate of CC.

## Conflict of Interest

The authors of the study declare there were no conflicts of interest.

## References

[R1] Thun MJ, DeLancey JO, Center MM, Jemal A, Ward EM (2010). The global burden of cancer: priorities for prevention. Carcinogenesis.

[R2] Torcia MG (2019). Interplay among Vaginal Microbiome, Immune Response and Sexually
Transmitted Viral Infections. Int J Mol Sci.

[R3] Chen W, Zheng R, Baade PD, Zhang S, Zeng H, Bray F, et al (2016). Cancer statistics in China, 2015. CA Cancer J Clin.

[R4] Shvartsman E, Hill JE, Sandstrom P, MacDonald KS (2023). Gardnerella Revisited: Species Heterogeneity, Virulence Factors,
Mucosal
Immune Responses, and Contributions to Bacterial Vaginosis. Infect Immun.

[R5] Norenhag J, Du J, Olovsson M, VerstraelRoleen H, Engstrand L, Brusselaers N (2020). The vaginal microbiota, human papillomavirus and cervical
dysplasia: a
systematic review and network meta-analysis. BJOG.

[R6] Pańczyszyn A, Boniewska-Bernacka E, Głąb G (2020). Telomere length in leukocytes and cervical smears of women with
high-risk
human papillomavirus (HR HPV) infection. Taiwan J Obstet Gynecol.

[R7] Manley KM, Luker R, Park C (2020). An audit of liquid-based cytology samples reported as high-risk
human
papillomavirus and borderline nuclear change in endocervical cells. Cytopathology.

[R8] Wright Jr, Cox JT, Massad LS, Carlson J, Twiggs LB, Wilkinson EJ (2003). 2001 Consensus guidelines for the management of women with
cervical
intraepithelial neoplasia. J Low Genit Tract Dis.

[R9] Chao X, Sun T, Wang S, Tan X, Fan Q, Shi H, et al (2020). Research of the potential biomarkers in vaginal microbiome for
persistent
high-risk human papillomavirus infection. Ann Transl Med.

[R10] Lv P, Zhao F, Xu X, Xu J, Wang Q, Zhao Z (2019). Correlation between Common Lower Genital Tract Microbes and
High-Risk
Human Papillomavirus Infection. Can J Infect Dis Med Microbiol.

[R11] Bhatla N, Singhal S (2020). Primary HPV screening for cervical cancer. Best Pract Res Clin Obstet Gynaecol.

[R12] Thomason JL, Gelbart SM, Wilcoski LM, Peterson AK, Jilly BJ, Hamilton PR (1988). Proline aminopeptidase activity as a rapid diagnostic test to
confirm
bacterial vaginosis. Obstet Gynecol.

[R13] Chacko MR, Kozinetz CA, Hill R, Collins K, Dunne M, Hergenroeder AC (1996). Leukocyte esterase dipstick as a rapid screening test for
vaginitis and
cervicitis. J Pediatr Adolesc Gynecol.

[R14] Calderón E, Rivera R, Gordillo S, Conde-Glez C (1997). Evaluation of a Fast Test to Identify the Presence of Proline
Aminopeptidase in Women With Bacterial Vaginosis. Infect Dis Obstet Gynecol.

[R15] Weston G, Dombrowski C, Harvey MJ, Iftner T, Kyrgiou M, Founta C, et al (2020). Use of the Aptima mRNA high-risk human papillomavirus (HR-HPV)
assay
compared to a DNA HR-HPV assay in the English cervical screening programme:
a
decision tree model based economic evaluation. BMJ Open.

[R16] Vos RA, Pasmans H, Tymchenko L, Janga-Jansen AV, Baboe-Kalpoe S, Hulshof K, et al (2020). High seroprevalence of multiple high-risk human papillomavirus
types among the general population of Bonaire, St. Eustatius and Saba,
Caribbean Netherlands.. Vaccine..

[R17] Castle PE, Varallo JE, Bertram MM, Ratshaa B, Kitheka M, Rammipi K (2020). High-risk human papillomavirus prevalence in self-collected
cervicovaginal specimens from human immunodeficiency virus (HIV)-negative
women and
women living with HIV living in Botswana. PLoS One.

[R18] Santella B, Schettino MT, Franci G, De Franciscis, Colacurci N, Schiattarella A, et al (2022). Microbiota and HPV: The role of viral infection on vaginal
microbiota. J Med Virol.

[R19] Wei ZT, Chen HL, Wang CF, Yang GL, Han SM, Zhang SL (2020). Depiction of Vaginal Microbiota in Women With High-Risk Human
Papillomavirus Infection. Front Public Health.

[R20] Dareng EO, Ma B, Adebamowo SN, Famooto A, Ravel J, Pharoah PP, et al (2020). Vaginal microbiota diversity and paucity of Lactobacillus species
are
associated with persistent hrHPV infection in HIV negative but not in HIV
positive
women. Sci Rep.

[R21] Sangpichai S, Patarapadungkit N, Pientong C, Ekalaksananan T, Chaiwiriyakul S, Thongbor R, et al (2019). Chlamydia trachomatis infection in high-risk human papillomavirus
based
on cervical cytology specimen. Asian Pac J Cancer Prev.

[R22] Zhao S, Zhao XL, Hu SY, Wang Y, Remila R, Xu XQ, et al (2019). Comparison of high-risk human papillomavirus infection rate and
genotype
distribution between Han and Mongolian women. Zhonghua Liu Xing Bing Xue Za Zhi.

[R23] Cao M, Wang Y, Wang D, Duan Y, Hong W, Zhang N, et al (2020). Increased High-Risk Human Papillomavirus Viral Load Is Associated
With
Immunosuppressed Microenvironment and Predicts a Worse Long-Term Survival in
Cervical Cancer Patients. Am J Clin Pathol.

[R24] Kang HJ, Chu KB, Kim MJ, Park H, Jin H, Lee SH, et al (2020). Evaluation of CpG-ODN-Adjuvanted Toxoplasma gondii Virus-Like
Particle
Vaccine upon One, Two, and Three Immunizations. Pharmaceutics.

[R25] Nikouyan N, Farhadi A, Gorzin AA, Geramizadeh B, Okhovat MA, Seyyedi N, et al (2020). A fluorometric hybridization assay for detecting and genotyping
high-risk
human papillomavirus 16 and 18 in archival tissues of cervical specimens. Braz J Microbiol.

[R26] Chan PK, Cheung TH, Li WH, Mei YY, Chan MY, Yim SF, et al (2012). Attribution of human papillomavirus types to cervical
intraepithelial
neoplasia and invasive cancers in Southern China. Int J Cancer.

[R27] Kyrgiou M, Mitra A, Moscicki AB (2017). Does the vaginal microbiota play a role in the development of
cervical
cancer.. Transl Res.

